# Chemical approaches to explore ubiquitin-like proteins

**DOI:** 10.1039/d4cb00220b

**Published:** 2025-02-12

**Authors:** Reem Mousa, Dana Shkolnik, Yam Alalouf, Ashraf Brik

**Affiliations:** a Schulich Faculty of Chemistry, Technion-Israel Institute of Technology Haifa 3200008 Israel abrik@technion.ac.il

## Abstract

Chemical protein synthesis has emerged as a powerful approach for producing ubiquitin (Ub) and ubiquitin-like modifiers (Ubls) in both their free and conjugated forms, particularly when recombinant or enzymatic strategies are challenging. By providing precise control over the assembly of Ub and Ubls, chemical synthesis enables the generation of complex constructs with site-specific modifications that facilitate detailed functional and structural studies. Ub and Ubls are central regulators of protein homeostasis, regulating a wide range of cellular processes such as cell cycle progression, transcription, DNA repair, and apoptosis. Ubls share an evolutionary link with Ub, resembling its structure and following a parallel conjugation pathway that results in a covalent isopeptide bond with their cellular substrates. Despite their structural similarities and sequence homology, Ub and Ubls exhibit distinct functional differences. Understanding Ubl biology is essential for unraveling how cells maintain their regulatory networks and how disruptions in these pathways contribute to various diseases. In this review, we highlight the chemical methodologies and strategies available for studying Ubls and advancing our comprehensive understanding of the Ubl system in health and disease.

## Abstract 2

This review outlines the chemical toolbox for preparing Ubls and their modified analogs, offering researchers a valuable resource that encourages the use of these methods to explore Ubl biology and their role in health and disease.

## Graphical abstract

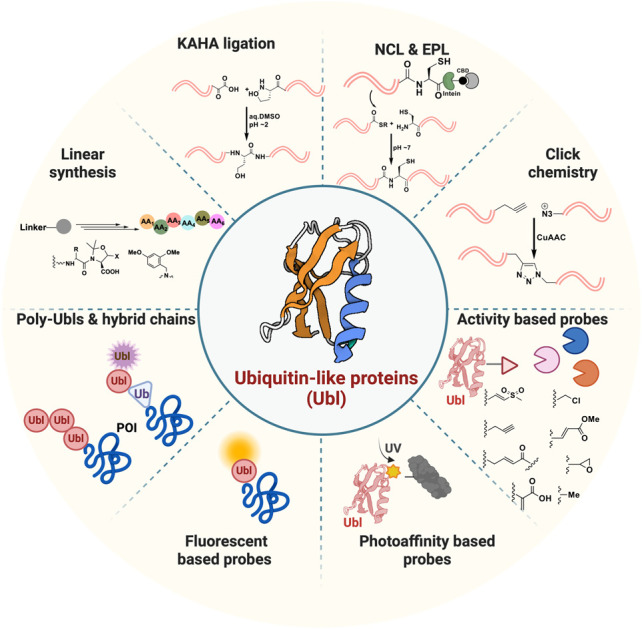

## Introduction

**Fig. 1:**
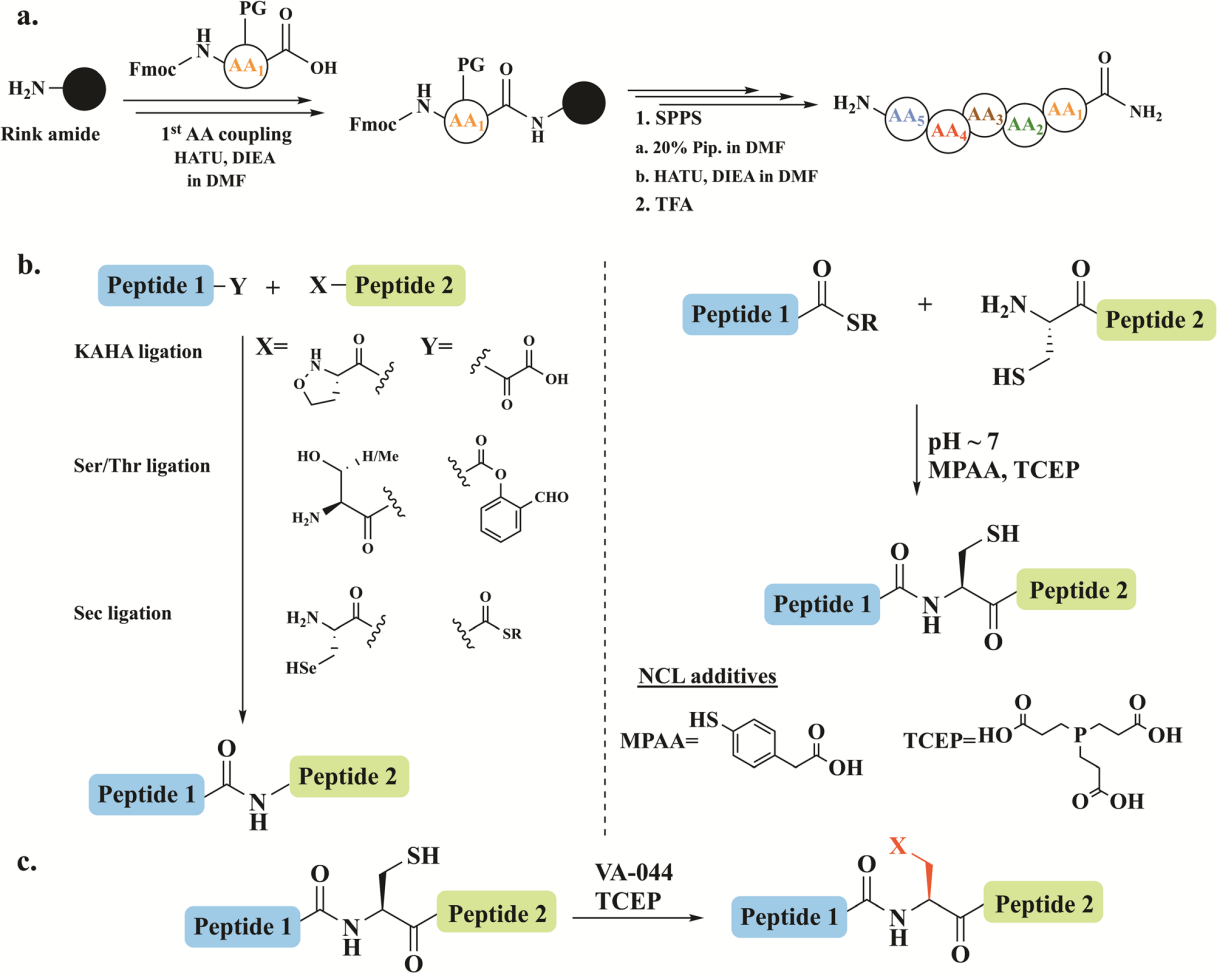
Schematic representation of (a) SPPS on a rink amide resin where activation, coupling, deprotection and cleavage steps are shown. (b) The different chemical ligation approaches, highlighting NCL. (c) The desulfurization reaction in the presence of the radical initiator 2,2′-azobis[2-(2-imidazolin-2-yl)propane] dihydrochloride (VA-044) and the reducing agent tris(2-carboxyethyl)phosphine (TCEP).

Chemical protein synthesis and semi-synthesis have emerged as revolutionary tools in protein research, enabling the precise preparation of proteins and their analogs with atomic-level control.^1–3^ Solid-phase peptide synthesis (SPPS) facilitates the assembly of peptides with defined sequences and modifications (Fig. 1a).^4^ When combined with chemoselective ligation methods such as native chemical ligation (NCL), it allows the generation of full-length proteins (Fig. 1b).^5,6^ In NCL, a C-terminal thioester of unprotected peptide is joined with an N-terminal cysteine/selenocysteine^7^ peptide under mild, aqueous conditions to form native amide bonds.

NCL has been further broadened by introducing desulfurization^8,9^ and deselenization^10–12^ reactions, which expands its applicability to the preparation of proteins lacking native cysteine residues (Fig. 1c). Additionally, other ligation strategies including serine/threonine^13^ and α-ketoacid-hydroxylamine (KAHA)^14^ ligation have expanded the scope of reactions to access diverse complex proteins (Fig. 1b).

Semi-synthesis further extends the capabilities of these methods by combining chemically synthesized fragments with recombinantly expressed protein domains.^15,16^ This hybrid approach enables the preparation of large proteins with different modifications, including non-canonical amino acids, isotopic labels, and post-translational modifications (PTMs). Chemical synthesis of proteins has enabled researchers to investigate their biochemical, structural, and functional properties in ways that are challenging to achieve using traditional molecular biology and enzymatic approaches. It also allowed the incorporation of specific and unique modifications to facilitate various studies such as the generation of activity-based probes (ABPs) designed to unravel a protein's interactome, expression level, and cellular localization.^1–3^

Chemical and semi-synthetic methods have been extensively applied to study ubiquitination and deubiquitination,^17–19^ key post-translational modifications that maintain protein homeostasis and regulate cellular processes.^20^ Ubiquitination involves attaching ubiquitin (Ub), a small, conserved protein with a β-grasp fold and a flexible C-terminal diglycine motif to substrates^21^ through an isopeptide bond with a lysine residue, facilitated by a cascade of E1, E2, and E3 enzymes.^22^ This process is counter reacted by deubiquitinating enzymes (DUBs) that remove Ub or Ub chains, modulating cellular signaling.^23–25^

**Fig. 2:**
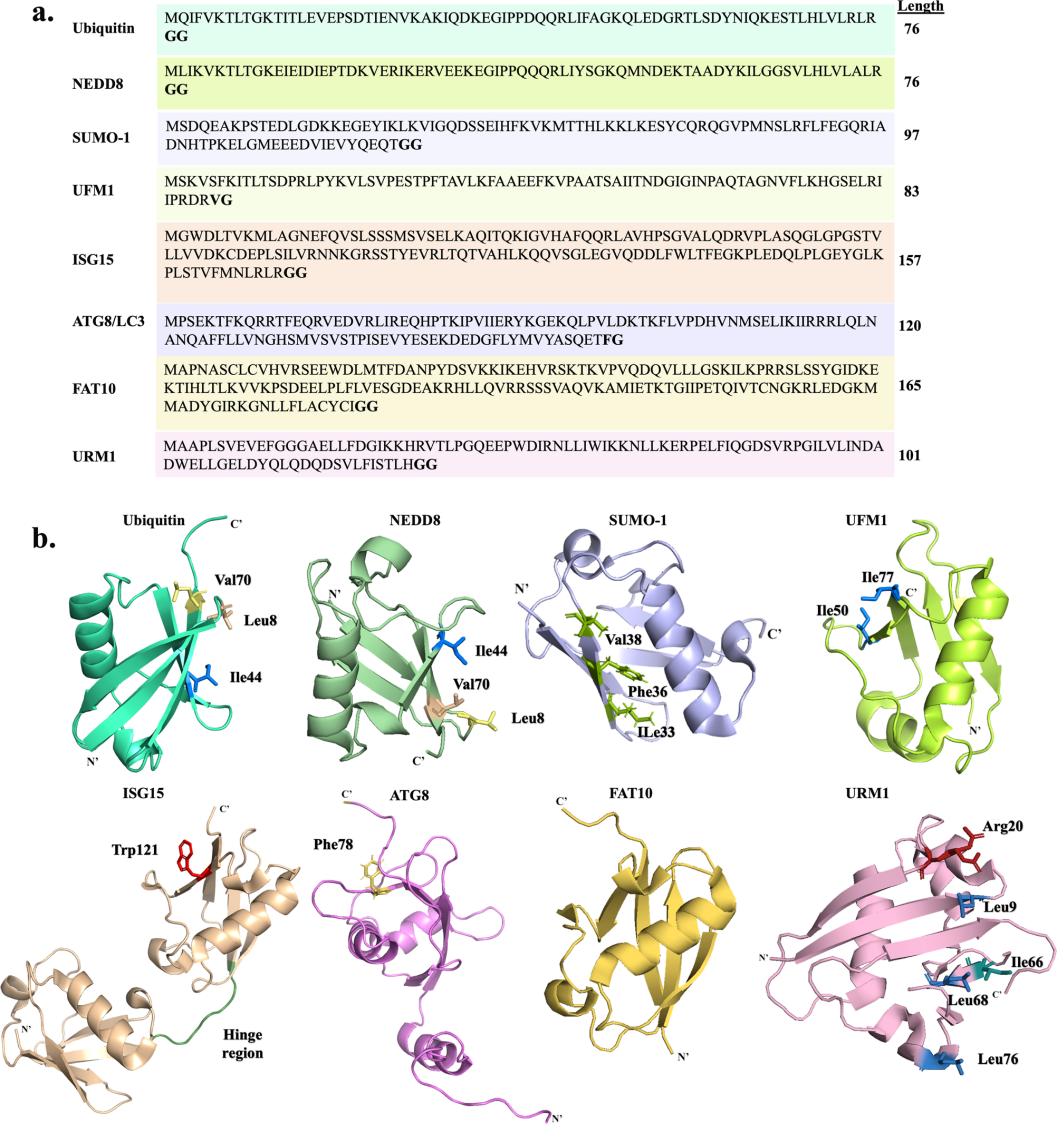
Representative conjugatable Ubl members. (a) Sequences of Ub and Ubls highlighting the diglycine motif and their length. (b) The tertiary structures of Ub and Ubls are also presented, highlighting several crucial structural elements (hydrophobic patches, Gly–Gly motif.). The PDB code for: Ub-1UBQ, NEDD8-1NDD, SUMO-1-4WJQ, UFM1-5IA7, ISG15-1Z2M, ATG8-2KQ7, FAT10-6GF1, and URM1-2QJL.

Ubiquitin-like proteins (Ubls), which are structurally similar to Ub, can also be conjugated to protein substrates *via* a similar mechanism, affecting various cellular processes.^26^ Humans have eighteen conjugatable Ubls, including five SUMO paralogs, NEDD8, UFM1, URM1, ISG15, ATG12, and FAT10, and seven ATG8 paralogs (Fig. 2).^27^ Research on Ubls focuses on their conjugation mechanisms, substrate recognition, specificity, interactions with other PTMs, and their roles in health and disease.^28^ Relying solely on enzymatic methods to prepare homogeneous Ubl-based conjugates presents challenges, such as limited availability of enzymatic machinery and difficulties in achieving site-specific modifications in sufficient quantities and homogeneity for biochemical and functional analyses. Chemical synthesis offers solutions to these challenges, enabling deeper exploration of Ubl biology and their involvement in various diseases.

In this review, we focus exclusively on UBLs that have been studied using chemical or semi-synthetic methods, except for FAT10, which has not been synthetically prepared. We highlight how the different approaches have contributed to our understanding of the various biochemical, structural, and functional aspects of Ubls. By providing this review, we offer a valuable resource for researchers to encourage them to use these methods to explore Ubls biology and understand their role in health and disease, potentially leading to new therapeutic applications.

## A brief overview

### SUMO

In humans, small ubiquitin-related modifiers (SUMOs) are a family of five small proteins ranging from 93–97 residues (Fig. 2a and b),^29–32^ which covalently modify their substrates in a process called SUMOylation, which regulates diverse cellular processes such as transcriptional regulation, DNA repair, and apoptosis.^33^ SUMO 1–3 are the most extensively studied paralogs and exhibit varying sequence homology, where SUMO-1 shares 18% homology with Ub and SUMO-2, and SUMO-3 shares 45% homology with SUMO-1.^29–32^ While Ub and SUMO share comparable enzymatic cascades, SUMOylation employs only one E2 enzyme *i.e.* Ubc9,^34,35^ while Ub has about 40 E2 enzymes (Table 1).^36^

**Table 1:** Summary of enzymes involved in Ubls conjugation and deconjugation and the chemical synthesis approache*s* used for their preparation in the free form or as conjugates

Family name	Enzymatic machinery			Proteases	Chemical synthesis approaches and applications
	E1	E2	E3		
SUMO family	SAE1	UBC9	RanBP2	SENP 1–3	NCL (SEA thioester): SUMO-1, SUMO-1-P53 peptide conjugate, SUMO-2, SUMO-3, SUMO-2 dimer, SUMO-3 dimer
SUMO-1	SAE2		PIAS 1–4	SENP 5–7	KAHA ligation: SUMO-2, SUMO-3
SUMO-2			ZNF451	DeSI-1,2	Direct SPPS (aggregation breaker): SUMO-2, SUMO-3
SUMO-3			Others	USPL1	NCL (Phcam linker): di-Ub(K63)-Lys11-SUMO-2, di-Ub(K63)-Lys33-SUMO-2 and di-Ub(K63)-Lys42-SUMO-2)
SUMO-4					Click chemistry: SUMO-1-RanGAP-1, SUMO-1-Ubc9, SUMO-2 -PML peptide conjugates
SUMO-5					
NEDD8	NAE1	UBC12	RBX1/2 DCN1	NEDP1	NCL ([Pd(allyl)Cl]_2_): NEDD8-cullin peptide
			Others	DEN1	KAHA ligation
				SENP8	Direct SPPS (backbone amide propargylation)
UFM1	UBA5	UFC1	UFL1	UFSP1	KAHA ligation
				UFSP2	
ISG15	UBE1L	UBCH8	HERC5	USP18	NCL
	UBA7		HERC6		NCL (Acm-NMe_2_): ISGylated-Ub
			EFP		
ATG8 family	ATG7	ATG3	ATG5	ATG4	EPL: lipidated-LC3
LC3A		ATG10	ATG12		
LC3B			ATG16		
LC3B2			Complex		
GABARAP					
GABARAPL1					
GABARAPL2					
ATG12					
FAT10	Uba6	USE1	Parkin	Not reported	Not reported
URM1	MOCS	?	?	Not reported	NCL (Cys alkylation to mimic Gln (Ψ-Gln))

**Fig. 3:**
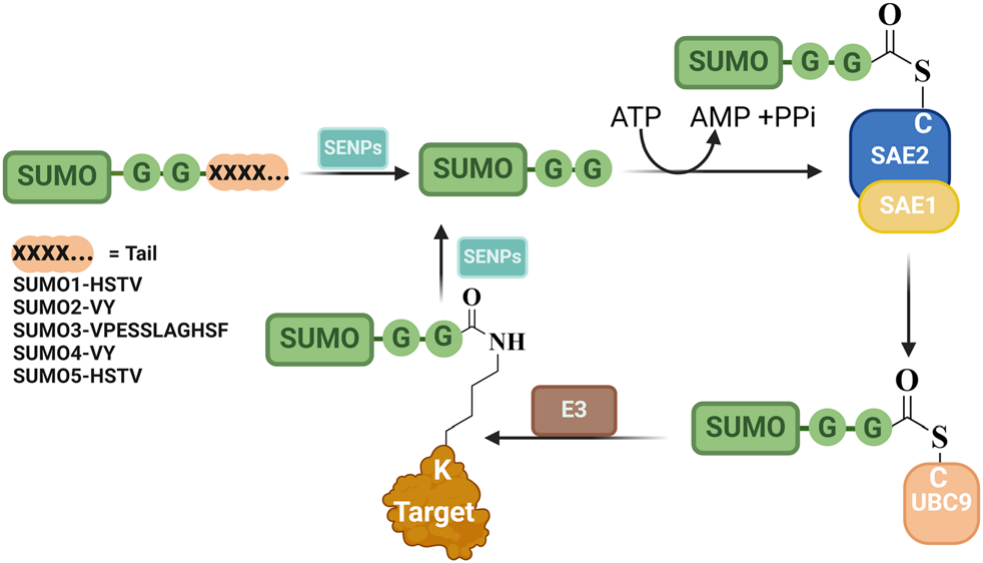
The reversible SUMOylation pathway, illustrating the covalent attachment and removal (deSUMOylation) of SUMO from its targets. This process comprises three key steps: E1 activation, E2 conjugation, and E3 ligation. The enzymes participating in each step are highlighted, along with the SUMO-specific proteases (SENP) responsible for deSUMOylation. Additionally, the distinct SUMO tails are depicted.

SUMO is conjugated to its substrates through an isopeptide bond between its C-terminal Gly and a substrate's Lys residue, leading to a single SUMO or poly-SUMO chain that is internally linked *via* an isopeptide bond(s) (Table 1 and Fig. 3).^37^ These modifications can alter protein stability, sub-cellular localization, and their intercoms. For instance, SUMOylation of RanGTPase-activating protein 1 (RanGAP1) targets it to the nuclear pore complex,^38^ while SUMOylation of promyelocytic leukemia (PML) assists in the assembly and stabilization of PML nuclear bodies (NBs), involved in DNA damage repair and antiviral responses.^39^

The Lys residue in substrates is typically located within a distinctive motif known as the SUMO consensus motif, featuring the sequence ΨKX(E/D), where Ψ represents hydrophobic amino acid, K is the modified lysine, X is any amino acid and E/D represents a negatively charged amino acid (either glutamate or aspartate).^40^ In addition to the covalent linkage, SUMO can interact with other proteins non-covalently through a SUMO-interacting motif (SIM) of a substrate.^41^

SUMOylation is known to be a reversible process where SUMO is cleaved from its substrate by SUMO-specific proteases (SENPs) (Table 1 and Fig. 3). SENPs are a family of cysteine proteases, comprising of seven members: SENP1–3 and SENP5–7.^42^ They are primarily localized in the nucleus, with certain members present in sub-nuclear compartments like the nucleolus and PML-NBs. SENPs play an additional role in the maturation process of SUMO where they cleave its tail, an extension of amino acids at the C-terminus, thus producing the mature SUMO with its di-Gly motif exposed and ready to initiate the cycle (Fig. 3).^42^ Since SUMO is involved in numerous cellular processes, aberration in SUMOylation can contribute to various diseases including cancer, neurological disorders, infections, diabetes, and others.^43^

### NEDD8

Within the family of Ubls, the 76 amino acid NEDD8 (neural precursor cell expressed, developmentally downregulated 8) has the highest sequence identity with Ub (∼59%), yet it possesses its own unique set of enzymes that ensure distinct conjugation pathways (Fig. 2 and Table 1).^44^

NEDDylation occurs *via* activation of NEDD8 by E1 (NAE1: APPBP1-Uba3 dimer), transfer to E2 (Ubc12), which is known to be highly specific and achieved by the presence of Ala72 in NEDD8 that allows the specific activation by the E1 and the consequent interaction of the N-terminus of E2 (Ubc12). The final step is a covalent conjugation to the substrate by an E3 ligase (RBX1, RBX2, and others).^45^

Like the majority of Ubls, NEDD8 is synthesized with a C-terminal tail that is cleaved by specific C-terminal hydrolases known as NEDP1, DEN1, SENP8, and the Ub hydrolase UCH-L3, to expose the diglycine motif, through which NEDD8 is covalently linked to different substrates.^46,47^ Like ubiquitination, NEDDylation is a reversible process where the proteases NEDP1/DEN1/SENP8 promote deconjugation of NEDD8 from its target (Table 1).^46,47^

The most well-characterized substrates for the NEDD8 are the cullin protein family, which serves as a scaffold for Ub ligase complexes and promotes ubiquitination and proteasomal degradation. The cullin family regulates proteins involved in the cell-cycle, transcription, signal transductions, regulation of O_2_, centrosomes, and cytoskeleton.^48–50^ NEDDylation can also modulate P53's stability by modifying its E3 Ub ligase Mdm2, leading to increased ubiquitination and degradation. The direct NEDDylation of P53 inhibits its transcriptional activity by prompting or inhibiting its ability to activate or repress target genes.^51^

### UFM1

The ubiquitin fold modifier 1 (UFM1) exhibits structural homology but lacks any obvious sequence identity with Ub.^52^ Composed of 83 amino acids (Fig. 2a and b), UFM1 is synthesized as an inactive precursor which undergoes maturation by two specific proteases – UFSP1 and UFSP2, to expose its C-terminal Gly residue.^53^ The covalent attachment of UFM1 to its substrates is termed UFMylation and occurs through an enzymatic cascade like Ub, activated by E1 (UBA5), transferred to an E2 (UFC1), and ligated to its substrate(s) by E3 (Ufl1) (Table 1).^53^ UFM1 can form polymeric chains through any of its six Lys residues, yet poly-UFMylation is reported to proceed predominantly *via* Lys69. Recent evidence suggests that UFMylation is involved in endoplasmic reticulum (ER) phagy, with findings showing that UFMylation on the ER surface acts as a signal for this process. In ER-phagy damaged or excess portions of the ER are targeted for degradation, ensuring the organelle remains functional and free of accumulated damage.

DDRGK1, an adapter protein of the UFMylation system, facilitates the recruitment of the UFMylation machinery to the ER surface for conjugation to various proteins embedded in the ER membrane such as RPN1, RPL26, and CYB5R3.^54,55^ UFMylation of these substrates induces recruitment of the ATG8 family, initiating autophagy of the UFMylated ER. Downregulation of UFM1-mediated ER-phagy leads to ER stress and accumulation of misfolded proteins.^55^

Like other Ubls, UFMylation is a reversible PTM, where UFSP2 is known to be involved in de-UFMylation. Loss of function of the UFM1 pathway is implicated in various diseases such as cancer, diabetes, schizophrenia, and ischemic heart disease. Additionally, it plays a crucial role in embryonic development and hematopoiesis due to its tight relationship with the ER stress response.^56^

### ISG15

Interferon-stimulated gene 15 (ISG15) is a small protein made of 157 amino acids that exists only in vertebrates, and is characterized by a unique structure wherein two Ub-like domains are linked together through a hinge region (Fig. 2a and b).^57,58^ ISG15 is initially synthesized as an inactive precursor which is proposed to undergo maturation to expose the C terminal Gly residue by two specific proteases: USP18, also known as UBP43, and the Ubp1-related protein.^59^ ISG15 is linked to its substrate through an ISGylation process that occurs *via* a known enzymatic cascade involving activation by E1 (UBA7/UBE1L), transfer to an E2 (UBCH8), and ligation to its substrate(s) by E3 (HERC5, HERC6, EFP).^60,61^ The reversibility of ISGylation is achieved by USP18, which is the only protease known so far for deISGylation (Table 1).

ISG15 serves a dual function: intracellularly as a protein modifier and extracellularly as a cytokine that is highly expressed and secreted upon IFN stimulation.^62^ As a protein modifier, ISGylation modulates various biological processes and displays an intricate interplay with ubiquitination. While in some cases upregulation of ISGylation inhibits Ub-mediated proteasomal degradation due to competition over Ub binding domains, in other cases, ISGylation can lead to Ub-mediated proteasomal degradation.^63,64^ Controversial results of the connection between ubiquitination and ISGylation suggest context-dependent outcomes. Moreover, while Ub-ISG15 hybrid chains are known to exist, their recognition by other proteins remains poorly understood (Table 1).

Notably, the expression of ISG15's E1, E2, and E3 is also highly induced by type 1 interferon (IFN), influenza B virus, lipopolysaccharide, and genotoxic stress. Aberration in ISGylation is associated with cancer, neurodegenerative diseases, and problems in response to pathogen infections, while normal ISGylation is crucial in embryonic development.^65^

### ATG8 and ATG12

Mammalians have six autophagy-related genes (ATG8 proteins) which are subdivided into the microtubule-associated protein light chain 3 – LC3 (LC3A, LC3B, LC3C/LC3B2) and GABARAP (GABARAP, GABARAPL1, GABARAPL2) members (Table 1). Each one of these proteins is composed of ∼120 amino acids (Fig. 2).^66^ The ATG8 C-terminus tail is cleaved by ATG4 protease to expose the Gly residue,^67^ and subsequently conjugated to its substrates through the cascade of E1-like ATG7, E2-like ATG3 and E3-like ATG12-ATG5:ATG16 complex (Table 1).^68^ Formation of the E3-like complex itself requires another Ubl conjugation pathway, where the Ubl ATG12 conjugates to the Lys residue of ATG5 in sequential reactions catalyzed by the specific E1 and E2. This conjugate further reacts with ATG16 to form the E3-like complex.^69^

ATG8 plays a crucial role in autophagosomal membrane formation, where its C-terminus is covalently linked to the phospholipid, phosphatidylethanolamine (PE), through the enzymatic cascade described before, forming a lipidated ATG8–PE.^70^ This lipidated form serves as a scaffold to recruit other autophagy-related proteins that are necessary for autophagosome formation. ATG8–PE also ensures the specificity and selectivity of proteins, organelles, and cellular components that are targeted for degradation.

This interaction is generally mediated by LC3-interacting regions (LIRs), located in the unstructured region of the ATG8-interacting proteins, and is composed of negatively charged amino acids followed by two hydrophobic amino acids spaced by two random residues.^70^ The attachment of ATG8 proteins to PE is reversed by the ATG4 proteases, which regulate its turnover on the autophagosomal membrane and the autophagy dynamics.^67^

Dysregulation of the ATG8–PE interaction is associated with various diseases such as cancer, infections, inflammation, and neurodegenerative disorders.^71^

### FAT10

Like ISG15, the human leukocyte antigen F-adjacent transcript 10 (FAT10) has a structure of two Ub-like motifs linked together through a hinge region^72^ (Fig. 2a and b), therefore it is also named diubiquitin or Ub D. FAT10 consists of 165 amino acids and is expressed as a mature protein that contains a free di-Gly motif necessary for conjugation (Fig. 2). While proteolytic activation is not required for FAT10, specific proteases may play roles in its deconjugations, yet no specific proteases involved in these processes have been reported.

The constitutive expression of FAT10 is restricted to immune system tissues, but its presence in other tissues can be induced by pro-inflammatory cytokines like interferon (IFN)-γ and tumor necrosis factor (TNF).^73^ Covalent attachment of FAT10 to substrates is termed FATylation and occurs through an enzymatic cascade like Ub, including activation by E1 (UBA6) transfer to an E2 (USE1) and ligation to its substrate(s) by E3 (suggested to be PARKIN) (Table 1).^74–76^ In addition to its involvement in immune responses and inflammation regulation, FAT10 directly targets its substrates for degradation by the 26S proteasome, making it unique amongst the Ubls.^77^ FAT10 is upregulated in various cancer types, such as gastrointestinal cancer, hepatocellular carcinoma (HCC), pancreatic ductal adenocarcinoma, and human glioma.^78^

### URM1

URM1 is among the least studied Ubls, also featuring the β-grasp fold and the C-terminal di-glycine motif (Fig. 2a and b).^79,80^ URM1 was identified through BLAST analysis for prokaryotic sulfur carrier proteins, noting its high sequence similarity to the proteins ThiS and MoaD.^81^ While URM1 shares similar structural features with Ub and other Ubls, it is expressed as a mature protein and is activated by an unusual mechanism leading to the formation of a unique C-terminus thiocarboxylate (–COSH).^82^ The URMylation pathway starts with the adenylation of the carboxylate (–COOH) by ATP-dependent E1 (Uba4 in yeast and MOCS in humans) forming an acyl disulfide bond. URM1 is then attached to a Lys residue on the substrate forming an isopeptide bond yet without evidence for the presence of E2 and E3 enzymes (Table 1).^83^ In addition to its role as a protein modifier, URM1 acts as a sulfur carrier essential for the 2-thiolation of wobble uridines (S2U34), a universal tRNA modification essential for coordinating translation and protein synthesis.^84^ In yeast, peroxiredoxin Ahp1 is the most studied substrate for URM1,^85^ suggesting its potential role in regulating cell redox status. This is further confirmed by the detection of 21 human proteins modified by URM1, under oxidative stress conditions.^86^

Recently, it was discovered that URMylation promotes the stress-dependent phase separation of target proteins aiding in stress resilience and cell survival.^87^ Since deUrmylases have not been identified yet, it remains unclear whether deURMylation occurs and if so, how this process might be reversed.

## Synthesis of Ubls and conjugates

Since Ubls in their free forms are relatively small proteins composed of 70–150 amino acids, they are accessible through chemical protein synthesis employing either direct SPPS or ligation approaches. Furthermore, combining these approaches with the semisynthetic one could also allow for the preparation of their conjugates. Here we describe, briefly, these methods and their application for the synthesis of various Ubls in their native or modified forms and their conjugates. Readers are also encouraged to peruse other comprehensive reviews of these methodologies.^1,2,18,88,89^

### Native chemical ligation (NCL)

**Fig. 4:**
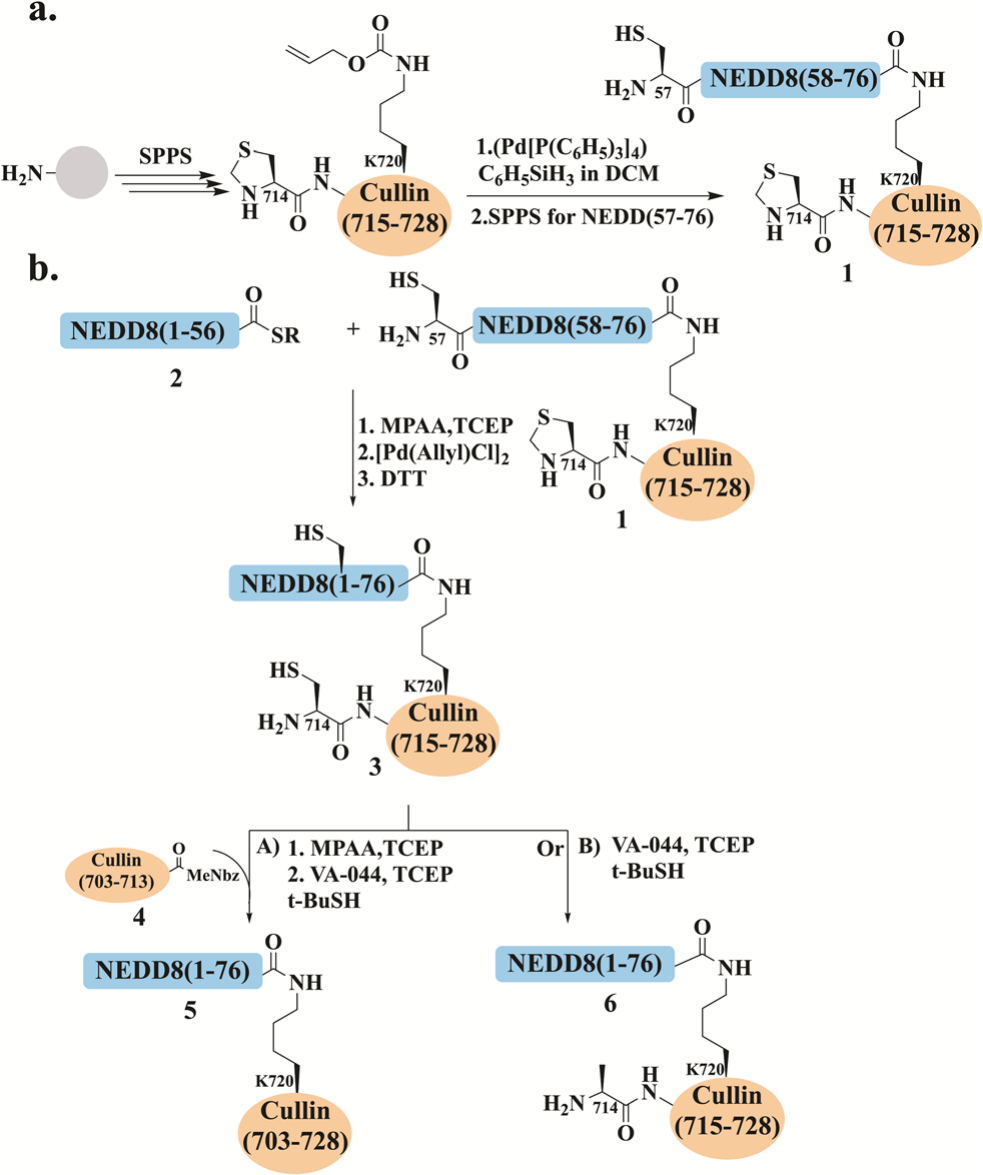
Schematic presentation for (a) the synthesis of NEDD8 (57–76) attached to cullin (714–728) through Lys720, where the alloc protecting group was incorporated during SPPS. Selective Alloc removal using the [Pd(allyl)Cl]_2_ complex allowed resin chain elongation of NEDD8 (57–76) to yield conjugate 1. (b) The preparation of NEDDylated cullin conjugate through NCL between (1) and (2), followed by Thz opening using [Pd(allyl)Cl]_2_. Direct ligation with cullin (703–713)-MeNbz (4) and desulfurization gave conjugate 5.

Since the introduction of NCL by Kent and his coworker, this method has been widely used to prepare hundreds of native and/or modified proteins. In this approach, chemoselective ligation of two unprotected peptides, one bearing a C-terminal thioester functionality and the other an N-terminal Cys residue, are ligated to form a native amide bond.^5^ Our group utilized NCL and desulfurization together with Pd chemistry to assemble a NEDDylated peptide derived from the cullin protein (Fig. 4).^90^

In this study, NEDD8 was prepared in its conjugated form with the 26-mer derived from cullin1 (703–728), a known substrate for NEDDylation. NEDD8 was prepared from two segments employing one ligation step at position 57, where Ala was mutated to Cys. First, the C-terminus of NEDD8 was prepared using Fmoc-SPPS where it was directly attached to Lys720 in the cullin1's C-terminus fragment on resin (Fig. 4a). This was achieved by using the alloc protecting group on Lys720, allowing for resin selective removal and peptide elongation to generate peptide 1 (Fig. 4a),^91^ which was ligated with peptide 2 to form the full-length NEDD8.

After assembly of NEDD8, the NEDDylated peptide was treated with [Pd(allyl)Cl]_2_, for thiazolidine (Thz) deprotection to form 3. This complex was demonstrated to be an excellent reagent for effective unmasking of Thz, enabling its removal within 15 min under NCL conditions.^90^ This intermediate was either ligated with the N-terminal peptide of cullin 4, followed by desulfurization to give the NEDDylated cullin conjugate 5 (Fig. 4b, path A), or directly deprotected and desulfurized to yield NEDDylated cullin conjugate 6 (Fig. 4b, path B). Conjugate 6 exhibited the secondary structure known for NEDD8 and was cleaved by the known ubiquitin C-terminal hydrolase isozyme 3 (UCH-L3).^92^ It should be noted that UCH-L3 is not the endogenous enzyme responsible for removing NEDD8 from cullins and was used solely as a model to provide evidence on the integrity of our synthetic conjugates.

**Fig. 5:**
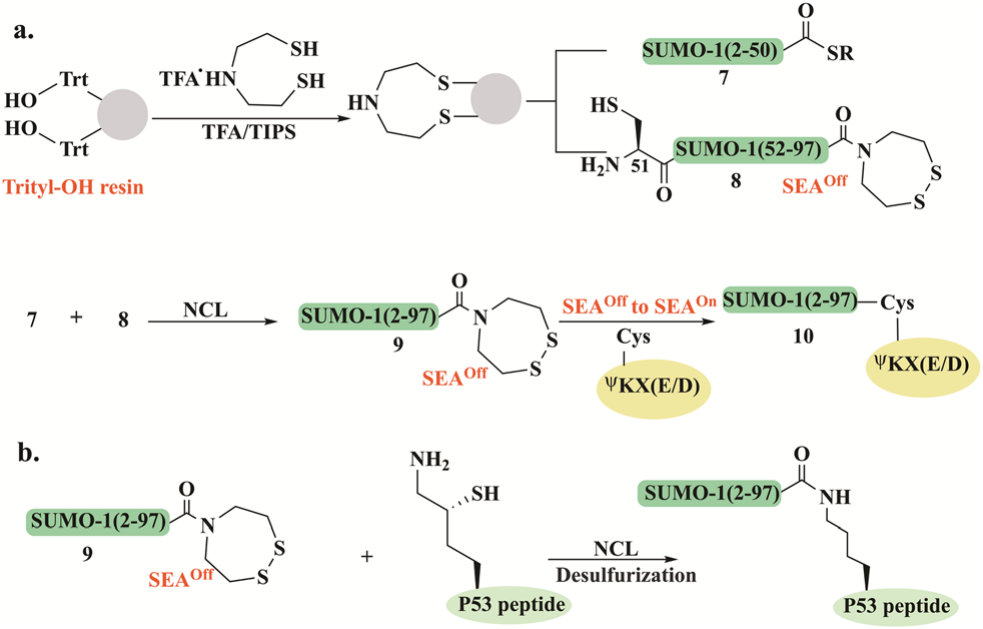
Schematic illustration for (a) the synthetic scheme of SUMO-1 conjugates starting from functionalized SEA-trityl-OH resin to synthesize 7 and 8 which further undergo sequential NCL reactions to form 10, and (b) the formation of SUMO1-P53 peptide conjugate based on the previous strategy.

Melnyk's group reported a synthetic approach for the preparation of SUMO-1, using the bis(2-sulfanylethyl) amido (SEA) thioester surrogate (Fig. 5a).^93^ SUMO-1(2–50) 7 and SUMO-1(51–97) 8 bearing a SEA^off^ (the cyclic disulfide form) were used for the assembly of full-length SUMO-1 9 (Fig. 5a). N- to C-sequential ligation was initiated by thioesterification of the SUMO-1(2–50)-SEA^off^7 by mercaptopropionic acid at pH 4 in the presence of TCEP, followed by NCL with SUMO-1(51–97)-SEA^off^8 to give 9. The full-length SUMO-1-SEA^off^9 was further activated to SEA^on^ allowing further attachment to a model peptide featuring the SUMO consensus motif ΨKX(E/D). The Lys residue in this peptide was modified with Cys to facilitate the SEA ligation with SUMO-1 (Fig. 5a). The folded domain of SUMO-1 in the synthetic conjugate 10 was confirmed by CD spectrum and the cleavage assay using Ulp1, a known Cys protease.

Using this strategy, the group prepared a SUMO1-P53 protein–peptide conjugate employing a one-pot approach based on three segments (7, 8, and P53 peptide). The SUMO-1-SEA^off^9 thioester was assembled using a single NCL reaction and further conjugated to P53 peptide through δ-mercapotlysine residue (Fig. 5b).^94^ To study the effect of Cys52 in SUMO-1 properties, the wild-type and Cys52Ala SUMO-1 analogs were prepared by selective and non-selective desulfurization, controlled by the absence or presence of denaturants in the reaction. These synthetic conjugates helped elucidate the important role of Cys52 in maintaining SUMO-1's structure, thermal stability, and functionality.

SEA linker was also employed for the preparation of SUMO-2 and SUMO-3 employing SEA-mediated ligation.^95^ The sequence homology and the inability to distinguish between SUMO-2 and -3 encouraged Melnyk and coworkers to investigate the role of the conserved Cys residue on SUMO-2 and SUMO-3 domain's stability and properties. Cys to Ala mutation was achieved by radical desulfurization under denaturing conditions. Both the secondary structure and the thermal stability analyses together with the conjugation and deconjugation studies revealed that mutating the conserved Cys47 in SUMO-3 must be considered with caution as the fold of SUMO-3 is significantly affected. Notably, this mutation interrupted the cleavage rate of the SUMO-3 conjugate by SENP1 and SENP2. This study highlighted that SUMO-2 and -3 are distinct proteins and should not be considered identical.

Following these studies, Ovaa and coworkers employed NCL to prepare another Ubl, ISG15. ISG15 was considered as a linear dimer of two Ub-like modules and therefore it was divided into two domains at the native Cys76 which were ligated to give the full-length ISG15.^96^

**Fig. 6:**
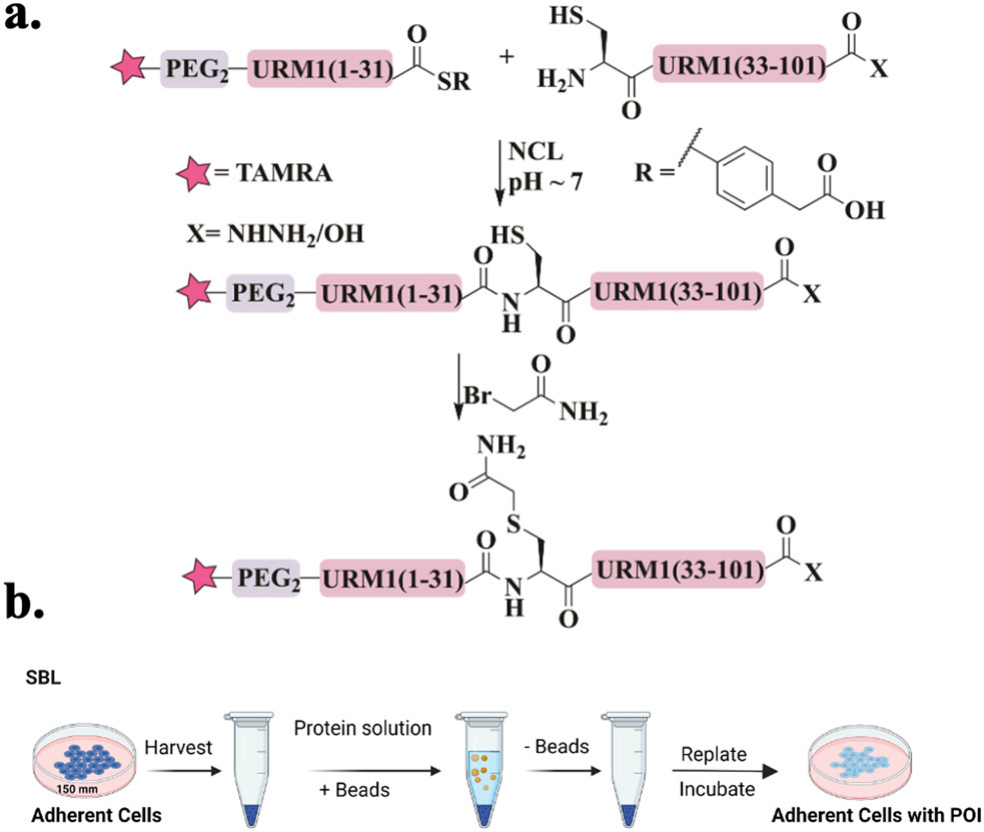
Schematic representation of (a) the URM1 synthetic approach. (b) The SBl delivery method utilizes glass beads to create physical disruptions in cell membranes, allowing protein molecules to enter suspended cells with minimal stress.

Recently, our group was able to access URM1 for the first time *via* a single NCL.^97^ Since URM1 lacks a Cys residue and its Ala residues are not suitably positioned for ligation and desulfurization, an alternative method was used. Glutamine at position 32 was substituted with Cys, which was then alkylated with bromoacetamide to produce pseudo-glutamine (Ψ-Gln), a mimic of Gln with a single atom difference (Fig. 6a). Using this approach three tetramethylrhodamine (TAMRA)-labeled URM1 analogs (Fig. 15c) were prepared containing different C-terminus modifications, carboxylic acid (URM1-COOH), hydrazide moiety (URM1-CONHNH_2_) and deleted glycine at position 101 (URM1-ΔG101-COOH). All URM1 analogs were delivered into cells using a newly developed method termed suspension bead loading (SBL), requiring only small quantities of protein compared to other delivery methods (Fig. 6b). As URM1 is a synthetically challenging protein, SBL provided an efficient, economical, minimally cytotoxic delivery platform.

We found that URM1 localizes mainly in the nucleolus under normal conditions and diffuses out in response to oxidative stress. Additionally, we have demonstrated that regardless of URM1's C-terminus, its localization and degree of conjugation are oxidative stress dependent.

### KAHA ligation

**Fig. 7:**
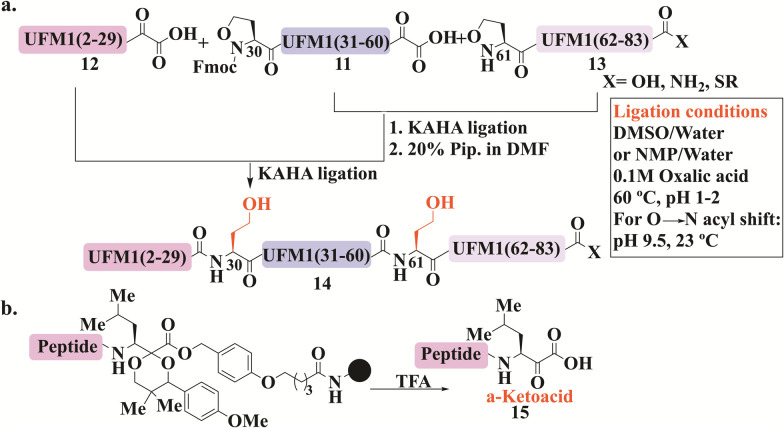
General schematic presentation for KAHA ligation applied for both UFM1 and NEDD8 syntheses, wherein (a) UFM1 was synthesized from three segments and two NCL reactions using Fmoc-Opr 11. The ligation conditions are also presented. (b) UFM1 synthesized similarly however, α-ketoacid 15 was prepared on resin.

KAHA ligation was developed by Bode and his coworkers to overcome the necessity of Cys (or thiol-modified amino acids)^8^ and a complementary thioester peptide in NCL.^98^ This ligation occurs between a peptide bearing α-ketoacids, and a peptide with N-terminal hydroxylamine, that undergoes chemoselective and reagent-less ligation. Substituted hydroxylamine, 5-oxaproline, was developed later to allow for effective ligation in acidic aqueous conditions. After ligation, the pH is adjusted to basic conditions, to facilitate an O- to N-acyl shift, leading to a homoserine residue at the ligation site (Fig. 7).^14,99^

KAHA ligation was used in the preparation of several medium-sized proteins, including Ubls. For example, UFM1 was the first Ubl to be synthesized using this ligation, where three analogs with different C-terminus modifications were prepared, carboxylic acid, amide, and thioester (Fig. 7a).^100^ These analogs were prepared from three peptides in two ligation steps, where the ligation sites were selected to be phe29-Thr30 and Ala60-Gln61. 5-oxaproline (Opr) was introduced for sequential KAHA ligation in peptides 11 and 13, where Fmoc protection was needed for the middle peptide 11 which was also equipped with α-ketoacids. The N-terminal peptide 12 was also synthesized with the α-ketoacid functionality. In addition, peptide 13 was functionalized differently at its C-terminus. Employing KAHA ligation between these fragments gave the desired protein 14 (Fig. 7a). CD analysis confirmed its secondary structure featuring multiple β-strands and α-helices. This synthetic method for UFM1, specifically the thioester analog, could potentially enable labeling with tags and site-specific conjugation to protein substrates.

Notably, the KAHA ligation process at its initial development required a key step where cyanosulfurylide had to be oxidized by oxone to form α-ketoacid. However, this step was incompatible with residues such as Cys, Met, and Trp, leading to undesired oxidation.^101^ Therefore, the group introduced a protected form of AA suitable for obtaining enantiopure peptide α-ketoacid 15 directly upon cleavage from resin (Fig. 7b). This method, which is compatible with all amino acids, was successfully applied to the preparation of SUMO-2/3 from three segments.^102^

Biochemical studies were performed to verify the structure and function of SUMO-2/3 that have homoserine residues due to the ligation requirement. Using SENP2, as a SUMO protease, successful cleavage of SUMO-2's tail to expose the di-Gly motif was observed. Additionally, the SUMOylation reaction on the substrate RanGAP1 demonstrated SUMO-2/3 activity. Both experiments illustrated that the homoserine residue does not affect the *in vitro* recognition and processing by the SUMOylation machinery.

**Fig. 8:**
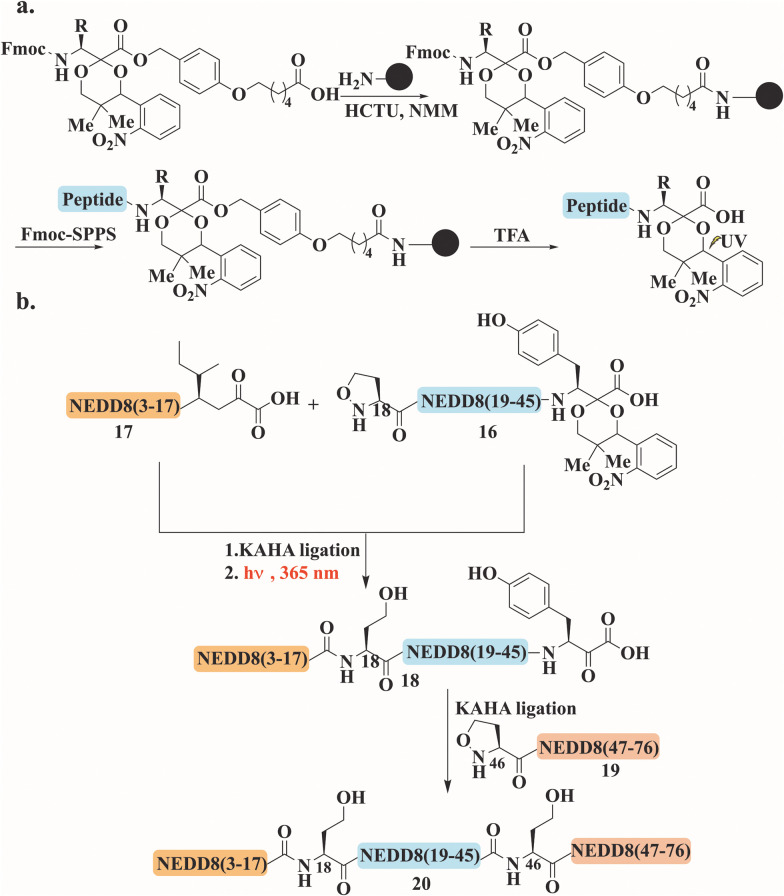
General schematic presentation for (a) the preparation of the photo-protected α-ketoacid and (b) NEDD8 synthesis through N- to C-NCL reaction, using three segments and two ligations where photo-protected α-ketoacid was introduced to peptide 16.

NEDD8 has also been accessed using KAHA ligation. Investigating the NEDDylation process and identifying new substrates has always been challenging due to difficulties in its expression. NEDD8 synthesis involved a newly developed photolabile protecting group incorporated in the α-ketoacid to facilitate one-pot multiple KAHA ligation (Fig. 8a).^103^ The photo-protected α-ketoacid in the desired peptide was unmasked under mild conditions through irradiation at 365 nm. Three distinct strategies were applied to prepare NEDD8, with two involving a three-segment process. The first strategy proceeded from the N-to-C-direction (Fig. 8b) using the photo-protected tyrosine α-ketoacid in peptide 16, which reacted with 17. The ligation product was directly irradiated to unmask the α-ketoacid. Peptide 18 was subsequently ligated with the C-terminal peptide 19, yielding NEDD8 (3–76) (20) (Fig. 8b).

The second approach operated from the C-to-N-direction using photo-protected oxaproline. It is worth noting, that in both strategies the segments were involved in sequential one-pot KAHA ligations including photocleavage of the appropriate protecting group under the ligation conditions. The third strategy is based on four segments applying three ligation steps and only one HPLC purification step. The synthetic NEDD8 was obtained in good purity and acceptable yield without intermediate handling or isolation steps.

### Expressed protein ligation (EPL)

**Fig. 9:**
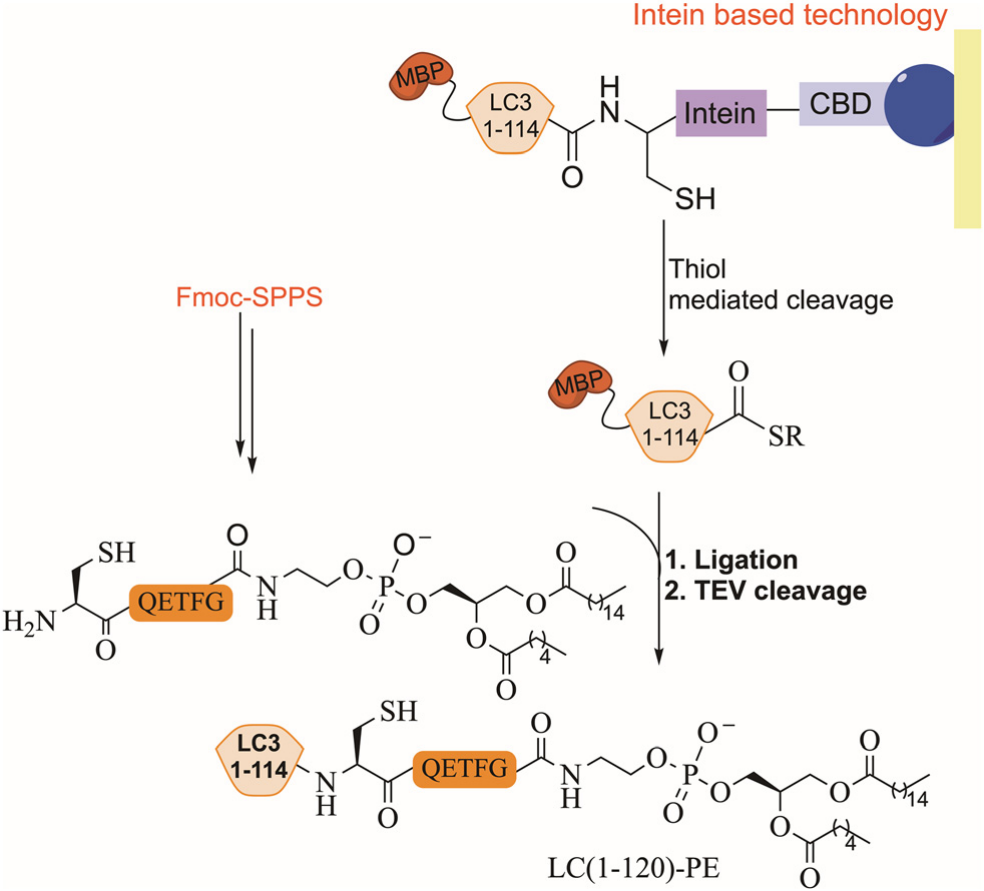
EPL strategy for the preparation of lipidated LC3, where intein based technology and Fmoc SPPS were used. CBD refers to the chitin binding domain and MBP refers to maltose-binding protein (cleaved by TEV).

EPL combines synthetic peptides with a recombinant large polypeptide often bearing a thioester moiety for the assembly of full-length proteins. The thioester fragment is obtained using intein technology while the synthetic peptide is prepared chemically and may contain various chemical modifications.^104^ Lipidated LC3 was prepared for the first time using a synthetic lipidated peptide and EPL to study its role in autophagosome formation.^105,106^ The LC3 fragment (1–114) was fused to an intein domain and a maltose binding protein (MBP), as a solubility tag, at the N-terminus (Fig. 9). Under folding conditions, the semisynthetic LC3(1–114)-thioester was ligated to the lipidated peptide in the presence of 4-mercaptophenylacetic acid (MPAA) as a thiol additive (Fig. 9), followed by the removal of the MBP tag using TEV protease.

*In vitro* activity of the semisynthetic LC3-PE was assessed using an ATG4 cleavage assay, demonstrating cleavage within 1 h. Furthermore, the function of LC3-PE in membrane tethering and fusion was also examined to underscore the significance of LC3 lipidation for membrane association and fusion promotion. Additionally, mutants at the C-terminus of LC3 assisted in understanding the structure–function relationship of the deconjugation specificities of ATG4 and RavZ proteases.^107^

### Direct SPPS

**Fig. 10:**
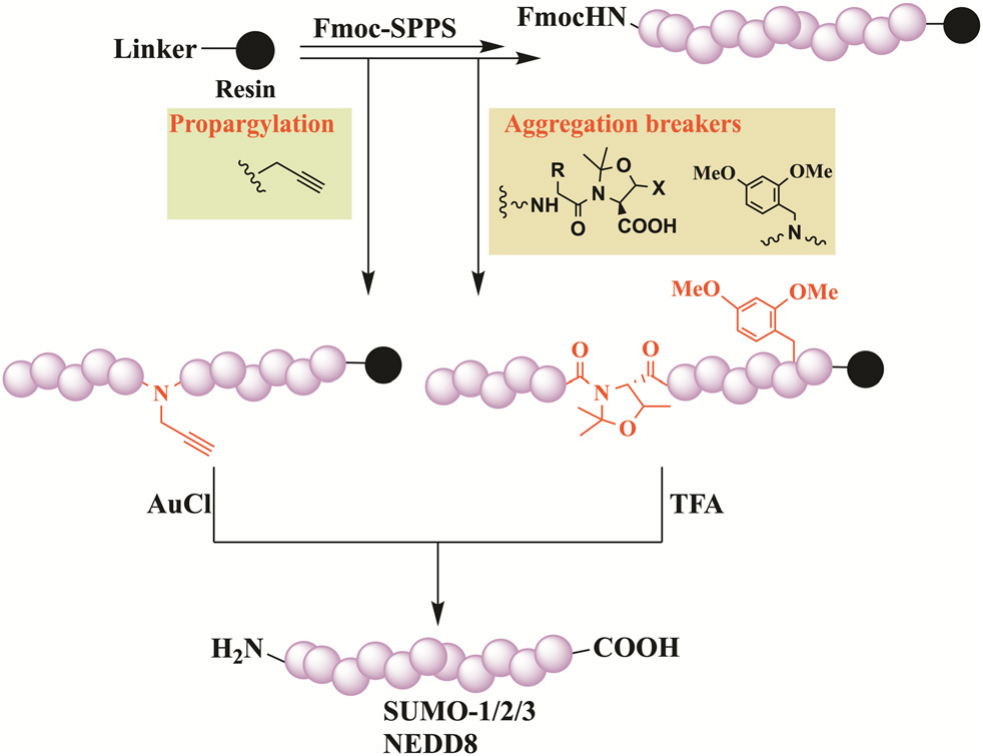
Schematic presentation of Fmoc-SPPS used for the preparation of SUMO-2 and SUMO-3 and for NEDD8 where aggregation breakers and backbone propargylation were introduced, respectively.

Although Fmoc-based SPPS is often limited to medium-length peptides (30–50 residues), Ovaa and his coworkers have succeeded in performing a direct SPPS to synthesize SUMO-1/2 and 3, without employing ligation approaches. Aggregation breakers such as pseudoproline and dimethoxybenzyl (DMB) were incorporated at different positions to improve synthesis (Fig. 10).^108^ All SUMO paralogs were obtained in very good purity, featuring the correct fold. NEDD8 was also synthesized without the need for ligation reaction and/or pseudoproline di-peptides. Instead, direct synthesis was employed (71AA), combining Fmoc-SPPS and backbone amide propargylation to act as a disrupting element, which is removed on demand using AuCl (Fig. 10).^109^

### Click chemistry

**Fig. 11:**
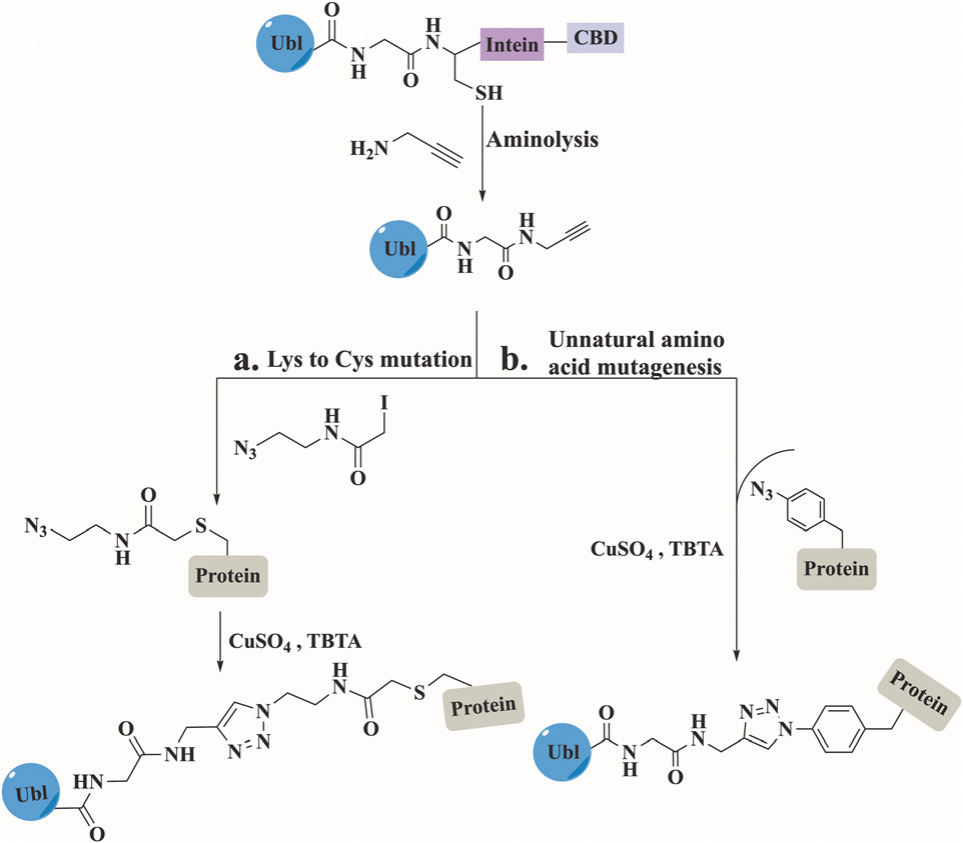
Preparation of modified proteins with Ubl, using click reaction between the expressed Ubls and the substrate obtained by (a) Lys to Cys mutation followed by iodoacetamide ethyl azide treatment or (b) unnatural amino acid mutagenesis.

Click chemistry between azides and alkynes using the copper(i)-catalyzed azide alkyne cycloaddition (CuAAC) reaction has been widely used for bioconjugation to prepare complex biomolecules.^110^ The Mootz's group has applied this chemistry to prepare three SUMO conjugates including, a short peptide derived from PML protein, a full-length Ubc9, and a fragment of human RanGAP1.^111–113^ In all conjugates, SUMO was recombinantly prepared to include an alkyne functionality at its C-terminus (Fig. 11). This was achieved by aminolysis of the intein thioester with propargylamine. The substrates including the azide functionality were prepared by either mutating the Lys to Cys that was treated with iodoacetamide ethyl azide, or by expressing the protein containing the unnatural amino acid. The click reaction was performed under non-denaturing conditions in the presence of CuSO_4_, TCEP and tris(benzytriazolylmethyl)amine (TBTA). Biochemical characterization of the SUMO conjugates demonstrated that the triazole linkage could serve as a stable mimic for the native iso-peptide bond (Fig. 11).

### Synthesis of poly-Ubls and hybrid chains

Although various methods have been developed for accessing polyubiquitin conjugates,^114,115^ the synthesis of poly-Ubl chains in their free form or linked to their native substrate(s) has not been well explored. Our group successfully synthesized, for the first time, a hybrid chain in which SUMO was linked to Lys63-di-ubiquitin. This hybrid chain has been reported to play a role in DNA double-strand break repair.^116^

**Fig. 12:**
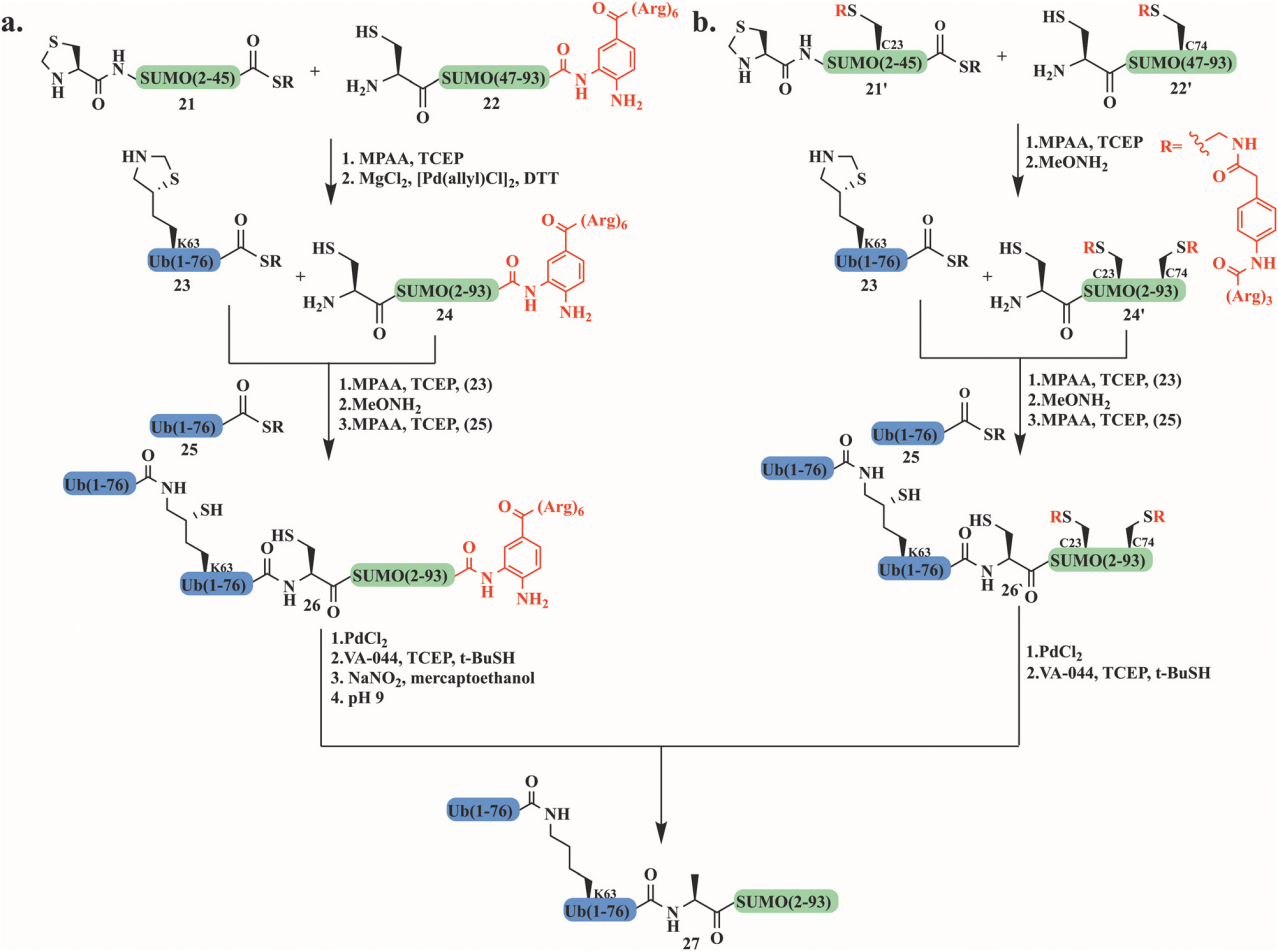
The synthesis of the hybrid chain SUMO-2-Lys63 linked-di-Ub introduced a polyArg solubilizing tag which was installed *via* (a) 3,4-diaminobenzoic acid (Dbz) or (b) the Phacm linker.

Using two different strategies we were able to synthesize four different SUMO-2-Lys63 linked-di-Ub. After failed attempts in conjugating the N-terminus of SUMO2 to Lys63-di-Ub, a polyArg tag was installed to 3,4-diaminobenzoic acid (Dbz) at SUMO2's C-terminus to increase the solubility and improve handling during the preparation and purification steps (Fig. 12a). Following two sequential ligation steps with the branched di-Ub and desulfurization, the tag was removed using three sequential steps that include NaNO_2_, thiolysis and hydrolysis to give the desired product 27. The second strategy, which turned out to be more effective, was based on attaching the polyArg tag *via* the phenyl-acetamidomethyl (Phacm) linker that was removed using PdCl_2_ upon synthesis completion (Fig. 12b).

Our strategy involved the synthesis of Thz-SUMO(2–45)-COSR (21′) and Cys-SUMO-(47–93) (22′) where two solubilizing tags were installed at Ala 23 and Ala 74 *via* the Phacm linker. The full SUMO (24′) was ligated to the first Ub-COSR (23), which was further reacted with another Ub-COSR (25) unit through δ-mercaptolysine.

The Di-Ub-(K63)-Cys-SUMO (26′) was subjected to PdCl_2_ to remove the solubilizing tag, followed by a desulfurization reaction to furnish the Ala native residues (Fig. 12b). Using this approach di-Ub(K63)-Lys11-SUMO-2, di-Ub(K63)-Lys33-SUMO-2 and di-Ub(K63)-Lys42-SUMO-2 were also prepared.

**Fig. 13:**
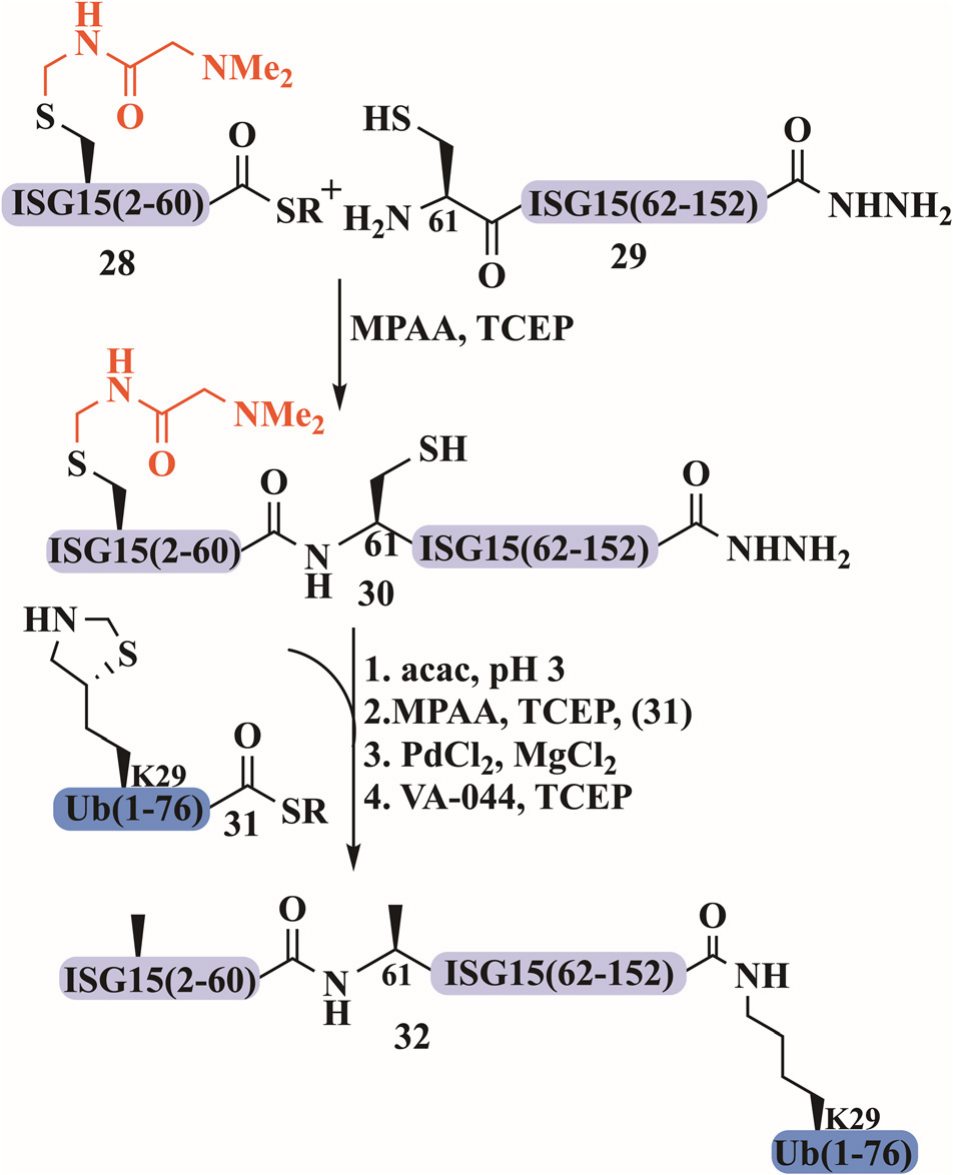
The synthesis of ISGylated-Ub hybrid chain using Acm-NMe_2_ as a solubilizing tag.

The Melnyk group also reported a rapid and robust synthesis for all SUMO-2/3 dimers to investigate how the composition of these chains impacts their properties.^94^ According to their strategy, SUMO-2/3 dimers were assembled through one-pot ligation between three segments. First, SUMO-2/3 was produced with a C-terminal SEA group, then ligated to additional SUMO-2/3 through Lys(Cys) at position 11. The secondary structure of SUMO-2/3 dimers was verified as well as their behavior with different SENPs, that confirmed their structural integrity. Additionally, our group prepared an ISGylated-Ub hybrid chain, where a new solubilizing tag, Acm-NMe_2_, was introduced during SPPS (Fig. 13).^117^ To overcome the hydrophobicity of the N-terminal region of ISG15, Ala residues at both positions 11 and 41 were mutated to Cys protected with an Acm-NMe_2_ tag. This modification was introduced to interfere with aggregation and facilitate solubility due to its charged tertiary amine. ISG15(2–60)-NHNH_2_ (28) and ISG15(61–157)-NHNH_2_ (29) were both synthesized by Fmoc-SPPS, where Cys78 at the hinge region was mutated to Ser to prevent dimerization, and Ala61 was mutated to Cys to enable NCL. Following ligation, ISG15 C-terminus 30 was activated to thioester using acetylacetone (acac) and MPAA to form the thioester, which was further ligated with Ub 31 *via* δ-mercaptolysine at position 29. The N-terminus of Ub was equipped with biotin and 6 Arg residues. The ISGylated Ub was then subjected to PdCl_2_ for Acm-NMe_2_ removal, to give product 32, which was extremely hydrophobic and difficult to handle (Fig. 13). Despite significant challenges in preparation and low purification yield, we were able to successfully characterize the natively folded conjugate by trypsin digestion, SDS-gel, western blotting, and CD measurement.

## Probes

### Activity-based probes

**Fig. 14:**
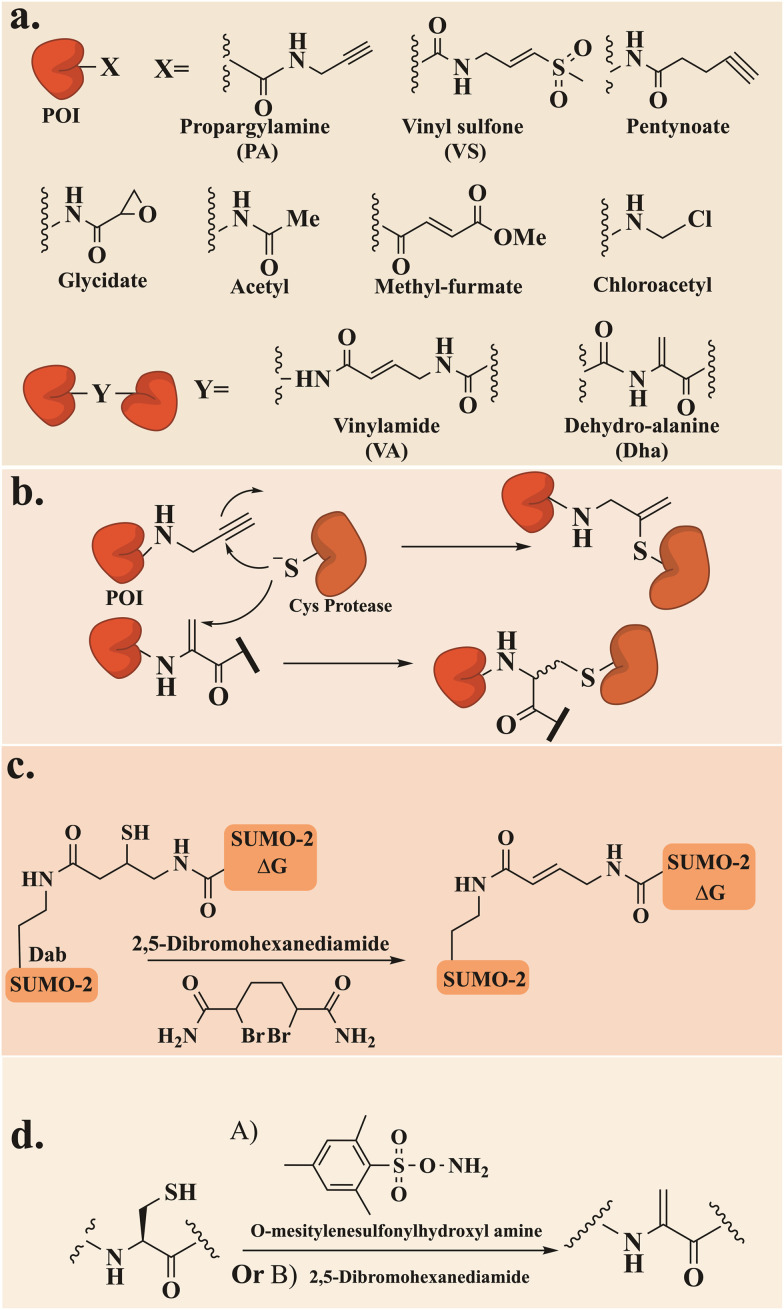
General scheme for (a) ABP chemical structures, (b) the chemical reaction between POI with PA and Dha and a Cys protease, (c) the preparation of di-SUMO bearing VA using a Dab residue and a ligation handle and (d) the preparation of the Dha probe using two approaches, A or B.

Activity-based probes (ABPs) have been used extensively to study the activity and function of Ubls in complex biological environments. These probes were designed to include a reactive warhead that interacts covalently with an active site of a target enzyme. Thus, enabling labeling and analysis of their functional state as well as their interactome. A C-terminal vinyl sulfone (VS) moiety was first introduced to NEDD8, ISG15, and SUMO-1 (Fig. 14a).^118^ The semisynthetic probe was prepared through intein-mediated expression to yield Ubls bearing a thioester, which was converted to vinyl sulfone using Gly-vinyl sulfone. The specificity of these probes toward purified conjugating or deconjugating enzymes *in vitro* was verified, where the VS moiety was involved in a Michael addition reaction with the catalytic Cys. Radioiodine labeling of Ubls-VS revealed a unique labeling pattern that reflects the distinct expression profile of active enzymes, indicating tissue-specific functions for Ubls. This allowed for identifying UCH-L1, DEN1, NEDP1, and SENP8 as a specific protease for NEDD8 and T/USP5 for ISG15, which were thought to be specific for Ub and SUMO. UCH-L1 and T/USP5 showed dual specificity with Ub/NEDD8 and Ub/ISG15.

The reactive warhead propargylamide (PA) was a useful probe for profiling SUMO-specific protases (Fig. 14a and b). Employing linear synthesis of SUMO, including *N-N*′-Boc-protected 5-carboxyrhodamine (Rho) dye together with PA, also allowed visualization of the SENP protease activity in cells.^108^ Ovaa and his colleagues successfully validated the reactivity and specificity of SUMO-based probes both *in vitro* and in whole-cell lysate. All SUMO isoforms exhibited reactivity toward SENP1 and SENP2, while SENP6 showed a slight preference for SUMO-2/3, and SENP3 and SENP7 demonstrated a distinct preference toward SUMO-2/3 probes. Cell experiments demonstrated that in response to ectopically expressed SENP enzymes, the cellular distribution of SUMO-2 and SUMO-1 was altered.

PA was further employed with Rho-labeled ISG15 to trap the known deISGylase USP18 in cell lysates. In lysate, where FLAG-USP18 and catalytically dead USP18 were overexpressed, only the active USP18 was labeled through its catalytic Cys.^96^ ABPs were further utilized to investigate the proteolytic cleavage of SUMO, synthesizing di-SUMO bearing vinyl amide (VA), which forms a covalent crosslink with the target enzyme (Fig. 14a).^108^ Linear Fmoc-SPPS employing diaminobutyric acid residue (Dab) and a ligation handle (4-((*tert*-butoxycarbonyl)amino)-3-(*tert*-butyl disulfaneyl)butanoic acid) at position K11 of the proximal SUMO-2 and thioester in the distal SUMO-2-ΔG, followed by NCL afforded the K11 diSUMO-2 precursor. Final thiol elimination with 2,5-dibromohexanediamide^119^ yielded the diSUMO2-VA (Fig. 14c). This unique probe participated also in both *in vitro* studies and in cell lysate to demonstrate its reactivity toward all SENPs, including the endogenous and ectopically expressed forms, except for SENP8 (NEDD8 specific protease). Additionally, introducing this probe revealed for the first time that SENP3 prefers diSUMO-2 over SUMO-1/2.^108^

The electrophilic group dehydro-alanine (Dha) has been employed to study the activity of Cys proteases, extending beyond the Ub system^120–122^ to include Ubls (Fig. 14a and b). Fluorescent UFM1 molecules, equipped with either Dha or PA reactive groups, were chemically synthesized by SPPS and NCL to capture conjugating enzymes and target cysteine proteases, respectively.^123^ Introducing Dha to the protected peptide was done by equipping the C-termini with Cys(Bn)-OMe which was later transformed by oxidative elimination with *O*-mesitylenesulfonylhydroxyl-amine to generate the UFM1-Dha probe (Fig. 14d). Rho-UFM1-Dha and Rho-UFM1-PA were applied *in vitro* and in cell experiments and their sub-cellular localization was visualized using confocal microscopy. UFM1-Dha showed reactivity toward the E1 conjugating enzyme UBA4, but no cross-reactivity with Ub conjugating enzyme UBE1. UFM1-PA was recognized only by UFM1-specific proteases, trapping Ufsp1 faster than Ufsp2. Introducing Rho-UFM1-PA by electroporation into either unmodified HeLa cells or those transfected with catalytically dead FLAG-Ufsp1 revealed colocalization exclusively with catalytically active Ufsp1 and distribution throughout the cell and nucleus.

The Dha probe has also been employed to study the semisynthetic ISG15, where it was expressed with a Cys to Ser mutation and with an additional C-terminal Cys.^96^ The probe effectively captured the established E1, E2, and E3, and the specific proteases USP18, USP5, and USP14, in various *in vitro* studies.

**Fig. 15:**
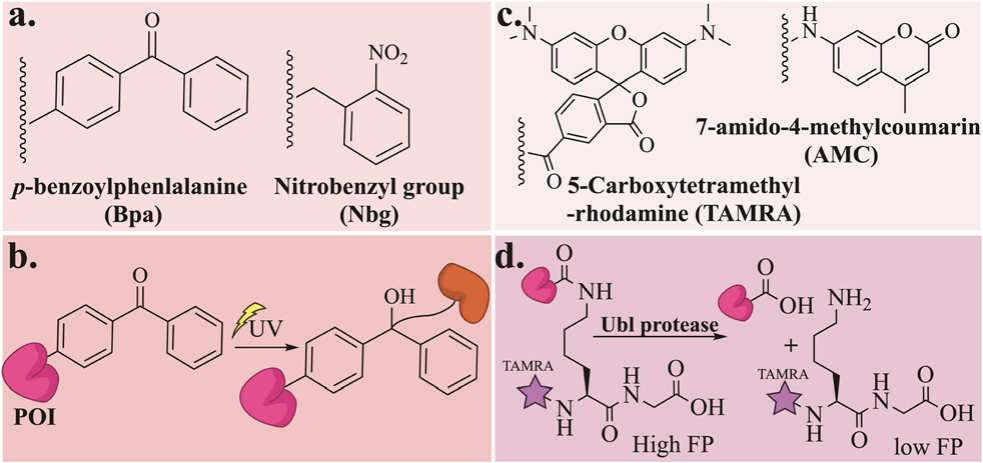
General scheme for (a) the chemical structure of the photoaffinity-based probe Bpa and the Nbg photocaged group, (b) the chemical reaction between POI loaded with the Bpa probe and an enzyme, (c) the chemical structure of TAMRA and AMC fluorophores and (d) the fluorescence polarization (FP) assay for the deconjugation of TAMRA-Ubl.

The production of Ubls with reactive groups was also achieved using intein-mediated recombinant peptide hydrazide. This hydrazide C-terminus serves as a handle for subsequent activation and aminolysis. The initial Ubl to be tested was UFM1-NHNH_2_^124^ which was oxidized with sodium nitrate, then subjected to aminolysis with propargylamide or thioesterification with MESNA, followed by aminolysis with glycine 7-amido-4-methylcoumarin to form the fluorescent probe UFM1-AMC (Fig. 15c).

This approach made ABPs accessible not only to chemistry laboratories but also to biological ones. The use of acyl hydrazide functionality was expanded by Bode and coworkers, who produced it through a single site-specific acetylation of recombinant UFM1-NHNH_2_ with different anhydrides at pH 3. This allowed access to the electrophilic warheads methyl-fumarate, glycidic acid, pentynoic acid, and acetic acid anhydrides derivatives (Fig. 14a).^125^

This method preserved the integrity of the folded protein and prevented reactions with unprotected amino acid side chains. These ABPs were used for *in vitro* and in-cell experiments. The chloroacetyl probe demonstrated remarkable selectivity towards the de-UFMylase UFPS2 in cell lysates and live cells (Fig. 14a). Following immunoprecipitation and proteomics analysis, only a small quantity of unspecific labeling was observed.

This method was further expanded to facilitated direct preparation of NEDD8 (ΔG76, ΔGG) and SUMO2 (ΔG93, ΔGG) ABPs, with different electrophiles without extensive preparation or purification steps.^126^ The specificity and cross-reactivity of NEDD8 and SUMO2 based probes with three known specific proteases USP21, SENP1, and SENP8, were investigated. These probes showed excellent specificity for their respective DUBs (SENP8 for NEDD8 and SENP1 for SUMO2), with minor cross-reactivity observed for NEDD8.

### Photoaffinity based probe

Photoaffinity probes have proven to be a powerful chemical tool for studying the non-covalent interaction between biomolecules with great detail.^127^ The probes incorporate photoactive groups that, upon activation by light, form a covalent bond with nearby amino acids. Photoaffinity probes were introduced to Ubls for the first time through the synthesis of di-SUMO2.^128^ The di-SUMO-2 was generated through hydrazide ligation of four synthesized segments, where the isopeptide bond was formed between Lys11 of distal SUMO-2 and Gly93 of the proximal SUMO-2. The probe was designed to include the photoaffinity group *p*-benzoylphenlalanine (Bpa) at Arg50 and a biotin tag at the N-terminus (Fig. 15a). UV irradiation at 360 nm for 10 min generated cross linked conjugates, as was observed in gel analysis, with different patterns for di-SUMO-2 compared to mono-SUMO-2 (Fig. 15b). The specificity of the probe was tested by examining the interaction between di-SUMO and RNF4, where a mutation in RNF4's SIM domain abolished the cross-link interaction. Furthermore, these cross-links were identified by affinity-based proteomics profiling to reveal a new binding protein specific for di-SUMO, RPS3, a protein involved in DNA damage repair.

Recently, the same group introduced on-demand photoaffinity SUMO-ABPs which were designed to capture SENPs *in vitro* and in live cells upon photo-irradiation.^129^ A bulky nitrobenzyl (Nbg) (Fig. 15a) photocaged group was introduced during the synthesis of SUMO-2-PA at Gly93 and at Gly64 to interfere with the binding with SENPs and prevent aspartamide formation, respectively. UV radiation at 365 nm cleaved the Nbg groups, granting SENP2 trapping *via* the propargylamine functionality. For in-cell SENPs profiling, SUMO-2 was labeled with a cell-permeable cR10 and D-biotin and delivered to HeLa cell, where SENP3 was successfully captured upon UV activation.

### Fluorescent based probe

Fluorescent probes were designed to enable biological and chemical research by visualizing, tracking, and quantifying molecules, and their interactions mainly in live cells. The fluorophore TAMRA, for example, has been extensively utilized in cellular protein studies (Fig. 15c), including research involving Ubls. TAMRA labeling of Ubls enabled not only tracking their distribution within cells but also analyzing their conjugation patterns using gel fluorescence. For example, ISG15-PA labeling allowed the trapping and detection of the deISGylation enzyme USP18,^96^ while SUMO-2 labeling facilitated the monitoring of its localization and conjugation patterns.^130,131^

The TAMRA fluorophore was also included in the development of a Ubl-fluorogenic polarization reagent to investigate the deconjugating activity toward various Ubl (Fig. 15d). Among these, a NEDD8–peptide conjugate was prepared by NCL between a synthetic NEDD8-thioester and TAMRA-labelled 5-thioLys-Gly using the E1 enzyme.^132^ The fluorescence polarization assay was used to test NEDD8 using a deconjugation assay with known UCH-L3 and USP21. UCH-L3 exhibited deNEDDylation activity, while USP21 showed no cross-reactivity toward NEDD8.

Fluorescence polarization was further applied to investigate the specificity of several SENPs. This was done using a high-throughput one-pot ligation desulfurization strategy for the synthesis of isopeptide-linked SUMO-3 in a 96-well plate, where each well contained a TAMRA-labeled peptide sequence of the active site of the most abundant SUMOylated proteins.^133^ The deconjugation assay was initiated by treatment with five different SENPs (SENP1, SENP2, SENP5, SENP6, and SENP7) resulting in a comprehensive dataset of SENP preferences for each SUMO substrate.

Recently, the acyl hydrazide functionality was applied to generate a fully folded fluorescence polarization substrate for Ub/Ubls. These substrates were activated from their C-terminal hydrazide to acyl-azide, followed by subsequent functionalization to isopeptides.^134^ Fully cleavable substrates (Ubl-KG-TAMRA) for SUMO1, SUMO2, NEDD8, and ISG15 were prepared with this procedure to investigate the substrate specificities toward human UCHL3, USPL1, USP2, USP7, USP16, and USP36. SUMO paralog's specificity toward USPL1 was re-investigated by fluorescence-polarization-based cleavage assays and rationalized with crystal structure analysis. For structural analysis SUMO-2/3 were prepared with either a 2-bromoethyl warhead or as a ΔN-SUMO2/3-PA(where the N-terminal domain was excluded) and covalently interacted with USPL1. Extensive biochemical analysis, including mutations at key residues of SUMO-2/3 at the interface with USPL1, revealed that the specificity of USPL1 toward SUMO-2/3 over SUMO-1 is attributed to its ability to recognize the Gly27 loop.

The applicability of the method was extended to NEDD8 and ISG15, which led to the discovery that USP16 and USP36 are active toward ISG15. Consequently, both USP16 and USP36 became the first human DUBs known for their specificity towards three distinct modifiers. Furthermore, the cross-reactivity of UCHL3 for NEDD8 and Ub was also reaffirmed in the same study.

## Conclusion remarks

In this review, we have highlighted the different methodologies and chemical approaches provided by the protein chemical toolbox to explore conjugatable Ubls. We have delved into the different synthetic approaches and the advances made in this field specifically for the preparation of Ubls in their free or conjugated forms as well as their probes. We have discussed the application of various chemical probes, such as ABPs, photoaffinity probes, and fluorescent probes, and how these tools have become instrumental in the understanding of Ubl biology. The chemical labeling strategies, combined with the design of Ubl-specific probes, fluorogenic proteins, and hybrid Ubl/Ub conjugates have enabled researchers to dissect the interplay between Ubls and their binding partners. These approaches enabled study of the Ubls’ real-time localization, signaling networks, and novel regulatory mechanisms, and identification of conjugation and deconjugation events.

The field still requires further expansion of the chemical toolbox to effectively access still difficult Ubl conjugates, particularly those that are highly hydrophobic and form longer chains on a particular substrate. Given the limited examples available in the literature regarding Ubls linked to their substrates or in their hybrid forms, our ability to replicate these complex biological systems and fully understand the critical aspects of Ubl chain regulatory mechanisms remains restricted.

Alongside more straightforward access to Ubls, we should also emphasize the need for advanced chemical techniques to study PTMs of Ubls,^135–138^ which are crucial for understanding how these modifications influence Ubl function and regulation, an aspect that remains to be fully explored. Moreover, we still need methods that allow us to follow the fate of proteins upon Ubl modification. The existing literature offers limited studies that investigate synthetic modified proteins introduced in their natural cellular environments, resulting in a limited understanding of these conjugates. With recent advances in the delivery of synthetic proteins to cells, it is now possible to take advantage of these precious synthetic conjugates to study their cellular behavior in cells. In particular, to study their localization under different cellular conditions, interactome and stability.

Additionally, delivery of external proteins could compensate for a lack of endogenous protein, thus rescuing the knockdown phenotype.

Towards these goals, we have recently synthesized SUMO-2 tail analogs, using the established synthetic methods and delivery approaches mentioned above, to examine their cleavage rate and cellular localization. The study revealed that the native SUMO-2 tail undergoes rapid processing and its critical role in nuclear localization and integration into PML-NBs.^139^

Innovations in probe design aimed at increasing specificity and sensitivity are also required to enhance our ability to study Ubls in diverse biological contexts, thus, enabling examination of their regulation machinery and aberration related to it. Finally, taking advantage of these conjugates and probes in therapeutic applications shows great promise, particularly in targeting Ubls and their related machineries in various diseases, similar to what has been done in the Ub system.^25,140^ Our recent work, where we identified *de novo* cyclic peptides through a combination of chemical protein synthesis and the RaPID system to modulate the Ub system, is one example of this avenue.^141–143^ These peptides exhibited strong selectivity for K48 or K63-linked Ub-chains, affecting their cellular behavior, including interactions with DUBs, the proteasome, and DNA repair machinery, positioning them as novel therapeutic candidates. Similarly, one could use any of the synthetic Ubl conjugates to find selective cyclic peptides to modulate biological processes.

## Author contributions

D. S. and Y. A. contributed to the literature search and wrote specific sections of the review article, while R. M. authored, reviewed, and edited the original draft, integrating all parts and designing tables, schemes, and figures for clarity. A. B. reviewed and edited the manuscript.

## Data availability

No primary research results, software or code have been included and no new data were generated or analysed as part of this review.

## Conflicts of interest

There are no conflicts of interest to declare.
